# A biotherapy based on PSCs-in-3D spheroid-ameliorated biologics depletes *in vivo* cancer-sustaining stem cells

**DOI:** 10.18632/oncotarget.5691

**Published:** 2015-10-19

**Authors:** Wenhui Zhang, Huanhuan Yang, Yanna Zhang, Yanan Lu, Tianlin Zhou, Meng Li, Yanjun Wen, Xiaojuan Lin, Rong Xiang, Xiancheng Chen

**Affiliations:** ^1^ National Key Laboratory of Biotherapy/Collaborative Innovation Center for Biotherapy, West China Hospital, Sichuan University, Chengdu, Sichuan, People's Republic of China; ^2^ Department of Gynecology & Obstetrics, West China Hospital/Second Hospital, Sichuan University, Chengdu, Sichuan, People's Republic of China; ^3^ Department of Immunology, Nankai University School of Medicine, Tianjin, People's Republic of China

**Keywords:** stem cells, 3D-spheroid, molecule microenvironment, immune renewal

## Abstract

CSCs are able to survive routine anticancer procedures and peripheral-immune attack. Here we develop and detail a framework of CSC elimination governed by 3D-biologics. Pluripotent cells-engineered 3D-biologics (PMSB) and control non-3D-biologics were prepared from placenta-based somatic stem cells (PSCs) and inoculated respectively into senile hosts bearing progressive mammary, lung, colon carcinomas and melanoma. We demonstrate that PMSB evokes *in vivo* central-immune microenvironment with subsequent re-expression of thymosin-α1 ~ β4 in thymic cortex-medulla borderline for rapid MHC-unrestricted renewal of γδT-dominated immunocompetence. The post-renewal γδT-subsets could accurately bind and drive CSCs into apoptosis. Finally, with central/peripheral integral microenvironment renewal and TERT/Wnt/β-catenin pathway blockade, the CSC-subsets are fully depleted, leading to substantial cure of diverse tumors by PMSB inoculation (*P* < 0.01), yet not by non-3D-biologics. Thus, our study may contribute to open up a new avenue for tumor remission via pluripotent cells-engineered 3D-biologics addressing quick renewal of central-thymus and peripheral immune-microenvironment.

## INTRODUCTION

Tumor progression and metastases are driven by the constantly evolving CSC subsets [1–2], a small population of self-renewing progenitor cells with capacity to render tumor resistant to peripheral-immune reactivity and conventional anticancer strategies including radiation, chemotherapy and small-molecule targeted therapies [3–4]. However, current antitumor therapeutics could stimulate peripheral defense reactivity only to amplified pool of terminal cancer cells, yet not to tumor-sustaining stem-pool resistant to routine therapies and MHC-restricted immune-recognition. Especially, modalities directing terminal cancer cells will boost cancer re-evolution to generate more resistant CSC-subsets due to Darwinian survival selection [5–6]. Where chemotherapy often removes the bulk of tumor mass without preventing tumor recurrence, and meanwhile chemotherapy might selectively enrich for hibernating CSCs, resulting in tumor re-evolution and relapse with ultimate death of the patient [7]. Thus, CSCs-directed therapy to resolve current anticancer dilemma remains a formidable clinical challenge. Nonetheless, just as infancy thymus provides superior cellular and humoral microenvironment as incubator of renewable T-cell subsets, so once retrogressive thymus microenvironment of tumor patients is equipped with special molecule re-expression, unique T-cell subsets may be reprogrammed for addressing *in vivo* resistant CSCs via evoking MHC-unrestricted immunocompetence renewal [8].

Human placenta-based somatic stem cells (PSCs), the sub-totipotent progenitors evolving from primary embryon towards somatic extraembryonic stem cells, have more primitive polyphenotypic features with greater multi-potentiality than bone marrow counterpart [9–11]. Embryonic stem cells have their totipotential to differentiate into any types of somatic cells; whereas PSCs could differentiate into mesenchymal, vascular, epithelial, neural, hepatic stem cells or other stem cells [12–16]. There are very similar biological features covering renewable/evolutionary dynamics and multipotent properties between PSCs and CSCs: *a*) high expression in embryogenesis antigen with low level of MHC-I molecules; *b*) stem-pool to escape from immune recognition; *c*) inducing the angiogenesis factors; *d*) limitless proliferative potential with active TERT; *e*) similar immunogenicity/reactogenicity; *f*) automatic reprogression mode with self-selected clonal heterogeneity/renewal [17–21]. In addition, PSCs ameliorated by X-ray could lead to phosphorylation and membrane translocation of calreticulin and further enhance cross-reactivity between PSCs and CSCs. Based on above informations, tumor-challenged senile hosts were intermittently implanted using PSCs-in-3D multipotent spheroid-ameliorated biologics (PMSB) to see if tumor development could be substantially impeded.

## RESULTS

### Establishment of PSC-3D-spheroids with multiepitope assay

**Figure 1:**
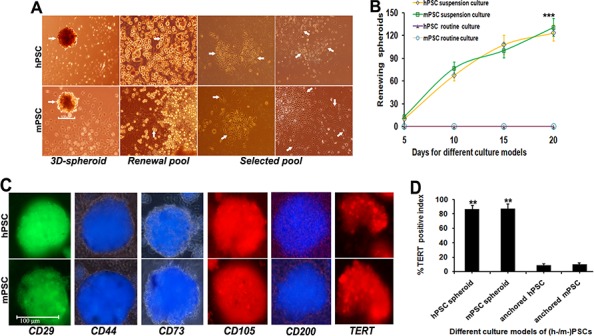
Selection and establishment of PSC-3D-spheroids **A.** 3D-PSCs were enriched via regenerating multicellular floating-spheroids; where each spheroid could enrich more than 220 stem cells bearing self-renewal and subset-evolution potential (3D-spheroid, arrow) with each dimension about 100–120 μm. PSCs address active self-replicating of round embryo-like stem cells (renewal pool, arrow) for self-renewal. PSCs regenerate various loose or dense grid-like patterns arranged with a range of cell lineages covering large polygonal, prismatic or cone-shaped cells; short or long spindle-shaped cells, asteroid or small polygonal cells (selected pools, arrows) with sequent subset-evolution and multiclonal-progression. **B.** Purified PSC populations were maintained in dynamic suspension for 20 days regenerating more than 125 floating 3D-spheroids/ml. Anchorage culture could not generate PSCs-3D-spheroids. **C.** PSCs-3D-spheroids share positive multiepitope-integration for CD29, CD44, CD73, CD105, CD200 and TERT with >90% synchronous expression demonstrated by dynamic spheroid-flow immunofluorescence scan. **D.** Computer-assisted histomorphometry revealed numerous TERT positive cells in PSCs-3D-spheroids, yet only a very few positive cells in common pattern of PSCs, with about 1/9 positive index of 3D-spheroids (*P < 0.05; **P < 0.01; ***P < 0.005).

PSCs were enriched via regenerating multicellular 3D floating-spheroids; where each spheroid could involve more than 220 stem cells with self-renewal and subset-evolution potential (Figure 1A arrow). PSCs address active self-replicating of round embryo-like stem cells for self-renewal (renewal pool, arrow). PSCs engaging subset-evolution loop regenerate a range of loose or dense grid-like patterns of heterogeneous stem cell lineages covering large polygonal, prismatic or cone-shaped cells; short or long spindle-shaped cells, asteroid or small polygonal cells (selected pools, arrows) with sequent multiclonal progression. Purified PSCs were maintained in dynamic suspension for 20 days regenerating more than 125 floating 3D spheroids/ml with each dimension about 100–120 μm (Supplementary Video S1–S2; Figure 1B). PSCs in anchorage culture could not generate 3D-spheroids (*P* < 0.005). Spheroid-flow immunofluorescence dynamic scan demonstrated that PSCs-3D-spheroids share positive multiepitope-integration for CD29, CD44, CD73, CD105, CD200 and telomerase reverse transcriptase (TERT) with over 90% synchronous expression (Figure 1C). Histomorphometry revealed numerous TERT positive cells in PSCs-3D-spheroids (Figure 1D), yet only a very few positive cells in PSCs under anchored culture pattern, with about 1/9 positive index of 3D-spheroids (*P* < 0.01).

### PMSB drives endogenous thymus renewal

**Figure 2:**
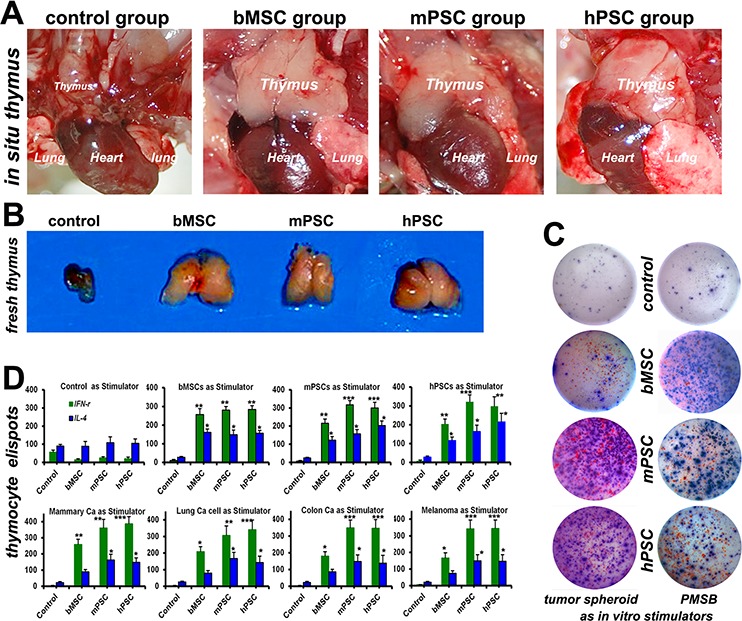
PMSB drives endogenous thymic renewal **A–B.** Withered senile thymus, just as shown in Control group, has revivified into renovation loop in PMSB (MSCs, m−/h− PSCs) groups and bulked over heart-size, with superior hyperemia facilitating thymopoiesis and active IFN-γ re-expression (C, red spots) in PMSB groups yet not in control group. **C.** Dual-color ELISpot assay confirms expression of IFN-γ (sustaining cellular immunity) and IL-4 (sustaining humoral immunity) by thymocytes from PMSB groups, yet not from control group, regardless of using PMSB or tumorsphere cells as stimulators, revealing central immune renewal in PMSB groups. **D.** PMSB *in vivo* inoculation could not only induce primary responses, but also trigger protective responses against *in vitro* tumors covering mammary, lung, colon tumors and melanoma spheroid cells (*P < 0.05; **P < 0.01; ***P < 0.005 versus Control).

The withered senile thymus (Figure 2AB) has bulked over heart-size with superior hyperemia in PMSB groups, with active IFN-γ re-expression (Figure 2C, red spots) of posterior thymopoiesis. Thymocytes from PMSB-formulated host thymus were used as responder cells and restimulated with PMSB or ray-modified tumorspheres including mammary, lung, colon tumors and melanoma for over 72 hours. Dual-color ELISpot assay reveals re-expression of IFN-γ and IL-4 by thymocytes from PMSB groups yet not from control group regardless of using PMSB or tumorspheres as stimulator, identifying central immune-renewal by PMSB. Histomorphometry via ELISpot plate reader confirmed that PMSB inoculation could not only induce primary responses, but also trigger protective responses against *in vitro* diverse tumors covering mammary, lung, colon tumors and melanoma spheroid cells (Figure 2D, *P* < 0.005 versus Control group).

### Integrative inspection for endogenous thymus renewal

**Figure 3:**
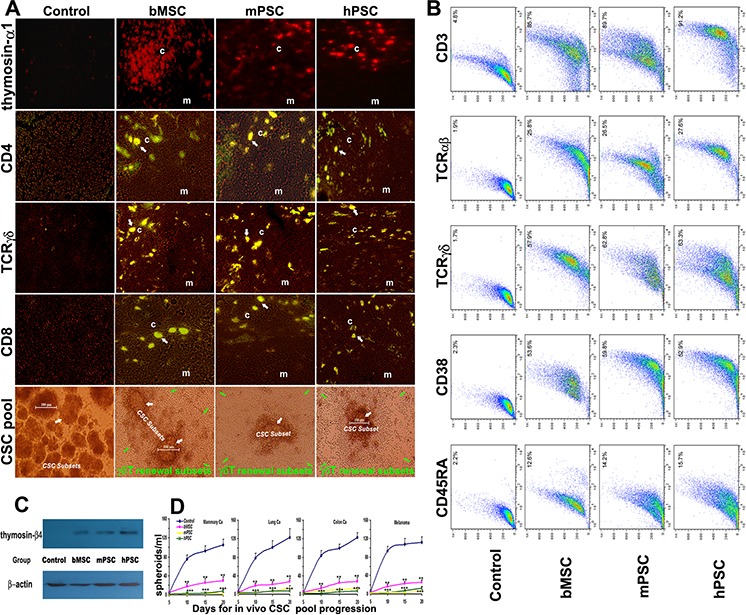
Integrative renewal of γδT-based immunocompetence **A.** Active thymosin-α1 re-expression (Panel 1) and plentiful thymopoiesis-renewal hotspots (Panels 2–4) in thymic cortex and cortex-medulla-borderline for CD4^+^/TCRγδ^+^CD8^+^ subsets undergoing repertoire renewal to remodel immunocompetence (arrows indicating borderline renewal hotspots) were revealed under confocal *in situ* scanning in PMSB groups, where cortical region (c) and medullary region (m) are indicated. Particularly, the renewable *in vivo* CSC-subsets (Panel 5, white arrows), enriched readily in control group, could be accurately bound and driven into apoptosis by the PMSB-renovated γδT-subsets (green arrows). **B.** T-cell repertoire renewal in PMSB groups has been verified by FACS assay, which shows the elevated expression index (*P* < 0.01 versus Control group) for CD3 (>85.7%), TCRαβ (>25.8%), TCRγδ (>57.9%), CD38 (>52.9%) and CD45RA (12.6 ~ 15.7%), with CD3 and TCRγδ manifesting high double peak subset elaboration in the repertoire renewal, therefore able to be responsible for addressing anterior immune-invisible CSC subsets under PMSB-renovated immune-microenvironment. **C.** Western blotting for thymic-extracted samples reveals active thymosin-β4 re-expression in PMSB groups, identifying central/thymic microenvironment renovation for MHC-unrestricted γδT quick-renewal. **D.** Insidious *in vivo* CSC pool in diverse tumors could be readily addressed by PMSB-based cellular-immunocompetence renewal (*P < 0.05; **P < 0.01; ***P < 0.005 versus Control group).

In thymic cortex and cortical-medullary borderline region of PMSB groups there are active thymosin re-expression (Figure 3A, Panel 1) and plentiful immunocompetent-subsets renewal hotspots (Panels 2–4) for CD4^+^/TCRγδ^+^CD8^+^ subsets to undergo redevelopment (arrows indicating the cortex and cortical-medullary borderline positive renewal hotspots), where cortical (c) and medullary (m) regions are indicated. Particularly, renewable *in vivo* CSC-subsets, enriched readily in Control group (Figure 3A, Panel 5, white arrows), have been encircled ring upon ring and inevitably eliminated by PMSB-remodeled MHC-unrestricted γδT-subsets (green arrows). T-cell repertoire renewal was verified by FACS assay, which shows the elevated expression index in CD3, CD38, CD45RA, TCRγδ and TCRαβwith double-peak elaboration for the diversity renewal (Figure 3B). Western blotting for thymic extracted samples reveals active re-expression of thymosin in PMSB groups, identifying central molecule-microenvironment renewal for MHC-unrestricted immunocompetence remodeling (Figure 3C). The experiment was repeated in four models with similar results (Figure 3D, *P* < 0.01).

### Magnetic bead microarray for peripheral molecular microenvironment

Magnetic bead microarray for extraction samples from non-necrotic fresh tumor or the tumor-free local inoculum identifies an enhanced elaboration of IFN-γ/IP10/RANTES loop in PMSB groups (*P* < 0.05), covering IFN-γ, IP-10 (IFN-γ-inducible protein 10, CXCL10), MIG (monokine induced by IFN-γ, CXCL9), CCL5 (regulated upon activation normal T cell expressed and secreted, RANTES). Concomitant IL-4 upregulation and VEGF downregulation were also demonstrated respectively in PMSB groups (Table 1).

**Table 1:** Molecule level elaboration of immune microenvironment

Molecules	Control	bMSC	mPSC	hPSC
IFN-γ (pg/ml)	21.02 ± 5.46	120.9 ± 21.1*	421.4 ± 53.9**	460.7 ± 60.5**
IP-10 (pg/ml)	506.2 ± 92.9	5212.1 ± 502.4**	10085.4 ± 933.8***	10103.5 ± 884.6***
MIG (pg/ml)	648.8 ± 77.1	10846.6 ± 1287.8***	31698.4 ± 2554.6***	32968.9 ± 3107.8***
CCL5(pg/ml)	35.2 ± 8.4	342.9 ± 40.7*	606.2 ± 54.9**	540.8 ± 83.9*
IL-4 (pg/ml)	5.76 ± 1.59	69.69 ± 9.1*	97.75 ± 9.03*	109.6 ± 11.5*
VEGF(pg/ml)	3434.5 ± 357.6	992.5 ± 89.4*	491.9 ± 67.8**	408.5 ± 30.6**

Values are expressed as means ± SD.

* *P* < 0.05

** *P* < 0.01

*** *P* < 0.005 versus control group, *n* = 8 mice per group.

Magnetic bead microarray for peripheral molecular immune microenvironment shows collective enhancement of key defense factors of IFN-γ/RANTES loop covering IFN-γ, IP-10 (IFN-γ-inducible protein 10, CXCL10), MIG (monokine induced by IFN-γ, CXCL9), CCL5 (regulated upon activation normal T cell expressed and secreted, RANTES) in PMSB groups (*P* < 0.05 versus control group, *n* = 8 mice per group), especially in two PSC groups. Integrative upregulation of IL-4 and downregulation of VEGF were also demonstrated.

### TERT/Wnt/β-catenin pathway blockade

CSC subsets from Control group readily re-generated hierarchical lineages including primary/renewing, evolving and selected/amplifying clones (subset progression/evolution). However, the subset-evolution properties have inevitably lost in PMSB groups (Supplementary Figure S2A Panel 1). Immunofluorescence manifested that active TERT, just as shown in control group, has declined evidently in PMSB groups (Supplementary Figure S2A Panel 2), with about 1/12 positive index of control group (Supplementary Figure S2B, *P* < 0.005 versus control groups). Active β-catenin expression, just as in control group, has declined or lost in PMSB groups (Supplementary Figure S2C). Namely, CSCs-governed TERT/Wnt/β-catenin loop has collectively vanished under PMSB-remodeled microenvironment.

### CSC progression deterred by PMSB-remodeled microenvironment

**Figure 4:**
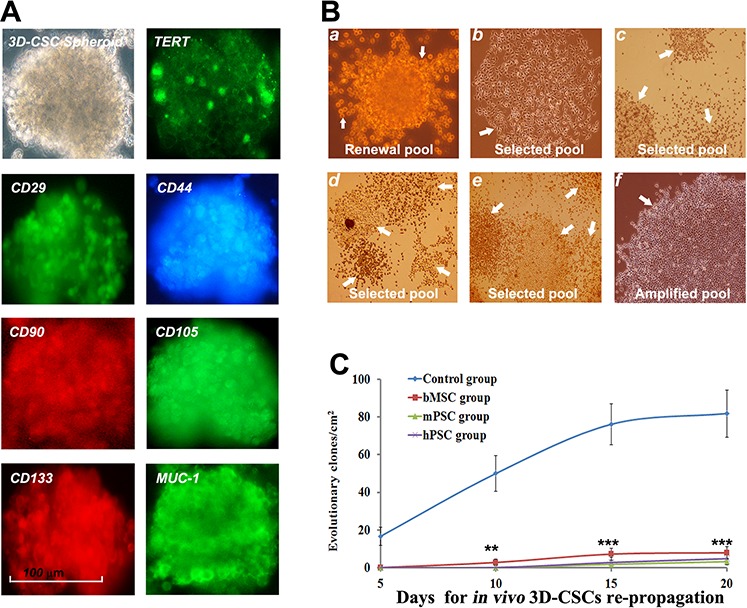
PMSB deters dynamic progression of CSC subsets **A.** 3D-CSC subsets from *in vivo* tumor of control group bear synchronous expression of TERT, CD29, CD44, CD90, CD105, CD133 and MUC-1 for renewing characteristics under 3D-spheroid-flow array. **B.** CSCs underlying self-renewal address active self-replicating of round embryo-like stem cells (a, renewal pool, arrow). CSCs underlying subset-selection comprise various loose or dense grid-like patterns arranged with a range of self-selected subsets (b, haploid evolving subset; c-e, multiple evolving subsets in CSCs selected pool) involving short or long spindle-shaped cells (b, c); large polygonal, prismatic and cone-shaped cells (d); asteroid or small polygonal cell clones (e). Bulk amplification and assembly of terminal tumor cells self-selected from subset evolution form new cancer nest for final tumorigenesis (f, the cancer nests in amplified pool of terminal cancer cells). **C.** Evolutionary index of 3D-CSC-subset from control group keeps progressive enhancement, with an average of 75 ~ 86 per cm^2^ at day 20 collectively. Yet evolutionary subsets hardly appeared in three PMSB groups respectively until day 15 and only very few appeared at day 20, meaning re-evolutionary ability of CSC pool distinctly retrogressed (*P < 0.05; **P < 0.01; ***P < 0.005 versus Control group).

3D-CSC subsets from *in vivo* tumor of control group manifest synchronous expression of TERT, CD29, CD44, CD90, CD105, CD133 and MUC-1 for renewable potential under spheroid-flow immunofluorescence scan (Figure 4A). CSCs address active self-replicating of round embryo-like stem cells for self-renewal (Figure 4B, a). CSCs share various loose or dense grid-like self-selected subsets (b-e) for subset-selection. Active amplification and assembly of terminal tumor cells self-selected from subset evolution mould new cancer nest for final tumorigenesis (f). 3D-CSC-subset dynamics assay indicated re-evolutionary index in control group keeping progressive enhancement, with an average of 75 ~ 86 evolutionary clones per cm^2^ at day 20 collectively. Yet evolutionary subsets hardly appeared in three PMSB groups respectively until day 15 and only very few appeared at day 20, thus re-evolutionary potentials of the CSC pool have distinctly retrogressed under PMSB-remodeled microenvironment (Figure 4C, *P* < 0.01 versus Control group).

### Elimination of migrating CSC subsets

**Figure 5:**
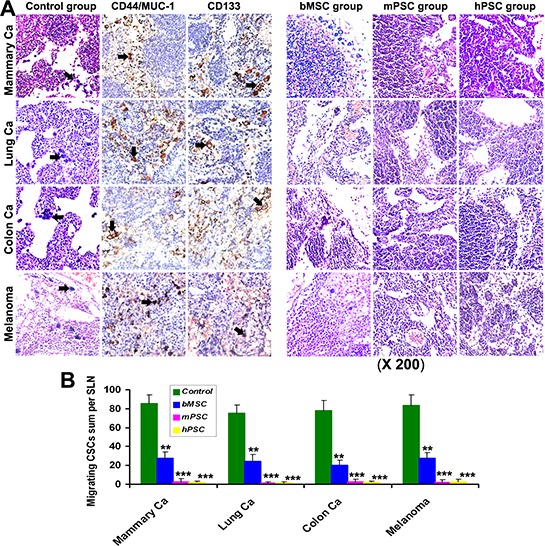
Abolition of migrating CSCs **A.** Detection for sentinel LNs prior to metastasis establishment indicated that there were many migrating CSCs (*arrows*, the self-selected subsets bearing metastatic potential with positive expression for CD44, CD133 and MUC-1) swimming in natural lymph fluid of *in vivo* dilative lymph sinus in Control groups. Yet such migrating CSCs have retrogressed or vanished in the lymph sinus of SLN in PMSB group. **B.** Histomorphometry demonstrated plentiful CSCs with migrating potential in the lymph sinuses of SLN in control group, yet only few or hardly such migrating CSCs in the SLN of PMSB groups (*P < 0.05; **P < 0.01; ***P < 0.005 versus control groups).

Prior to metastasis establishment, plentiful CSC-subsets (MUC-1^+^, CD44^+^, CD133^+^) with migrating potential were detected to drift from local tumor into the sentinel LNs (SLN) via natural lymph flux assay in dilated lymphatics/sinus in control group (Figure 5A). Yet such self-selected migrating CSCs have distinctly deterred or not survived in PMSB groups. Experiment was repeated with similar results in four different models covering mammary, lung or colon tumors and melanoma. Computer-assisted histomorphometry demonstrated plentiful CSCs with migrating potential in the lymph sinuses of SLN in control group, yet only few or hardly such migrating CSCs in the SLN of PMSB groups (Figure 5B).

### Collaborative renovation of local immune microenvironment

Tumor growth was evidently impeded in PMSB groups, with a growth inhibition rate of over 71% at 3 weeks and 68% at one month (Supplementary Figure S3A). All hosts in Control group developed tumors prior to day 12 post-tumor challenges; yet half of hosts in PMSB groups kept tumor-free survival in ultimate stage (Supplementary Figure S3B). Peripheral immune microenvironment analysis by histomorphometric and FACS assay shows that plentiful CD8^+^ and CD4^+^ T cells (CD25^−^Foxp3^−^) congregate in tumor stroma of PMSB groups. Whereas, numerous CD25Foxp3^+^ (positive index > 97%) T cells infiltrated intratumoral tissues and milieu in control group, but only few such T cells (<0.76%) in PMSB groups (Supplementary Figure S3C).

### Selective depletion of *in situ* CSC subsets

Since MUC-1 is a marker present on more than 90% of CSCs population covering colon, breast, ovary, prostate, and lung cancers [22], *in situ* MUC-1/NK-1 positive expressions of tumor were evaluated. Cancer nests in control group kept active proliferation with plentiful MUC-1^+^ tumor cells scattering and sparse NK-1^+^ lymphocytes surrounding tumor milieu (Supplementary Figure S4A). In contrast, the remnant cancer nests in PMSB groups have gradually retrogressed via cytolysis, karyorrhexis and pyknosis, with significant NK congregating and extensive macrophage infiltrating yet very sparse MUC-1^+^ cells surviving. Especially, the macrophages recruited to tumors in PMSB groups expressed high levels of CD80 (>81%) for M1 subset versus control group (<1%). Tumor cells MUC-1 positive index was very low in PMSB groups, with only about 1/9 ~ 1/10 of control group, indicating *in situ* CSC pool was selectively depleted (Supplementary Figure S4B). Meanwhile the lymphocytes NK-1 positive index in PMSB groups was fivefold over Control (Supplementary Figure S4C).

### IFN-γ/RANTES loop blockade boosts tumor progression

Blockade of key factors in IFN-γ/RANTES defense loop including IFN-γ, IP-10/CXCL10, MIG/CXCL9 and RANTES/CCL5 could accelerate tumor formation and progressive dynamics of PMSB-formulated hosts (Supplementary Figure S5AB). Tumor-free induction by PMSB were mostly reversed by IFN-γ or IP-10/CXCL10 blockade and evidently deterred by MIG/CXCL9 or RANTES/CCL5 blockade (*P* < 0.05 versus controls). Magnetic bead microarray also revealed that blockade of any one of these cytokines caused a concomitant decrease in the other cytokines, thus indicating the interdependence among them.

### Collaborative renovation of peripheral microenvironment with metastasis blockade

**Figure 6:**
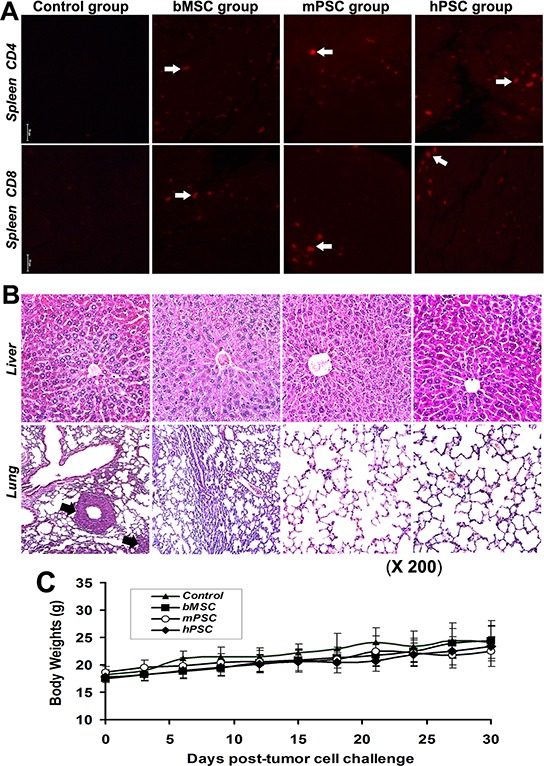
Renewal of peripheral immune microenvironment **A.** Immunofluorescence assay reveals numerous CD4/CD8 positive subsets (*arrows*) in spleens of PMSB groups (*P* < 0.01 versus Control group), indicating integrative resuscitation of peripheral defense loop following central immune renewal. **B.** No microscopic toxic and cell-deposited clinical or pathologic indications in livers and lungs are detected in PMSB-inoculated groups. No metastasis was found in the lungs of PMSB-inoculated groups; yet there are visible metastases around the lung blood vessels in over 90% of hosts of Control group (*arrows*, lower panel). **C.** Body weight curves of PMSB-inoculated groups keep paralleling closely that of Control group, with no significant differences among them. All animals thrive after PMSB vaccination, as well as no evidence of autoimmunity, indicating lack of toxicity-correlated weight loss in PMSB-inoculated hosts (*P* > 0.05).

Immunofluorescence assay reveals numerous CD4/CD8 positive cell subsets in spleens in PMSB groups (Figure 6A). No relevant adverse effects, in both macroscopic changes such as skin tenting or ulcerations, ruffled fur or toxic death, and microscopic signs such as toxic or cell-deposited pathologic lesions in heart, lungs, livers, kidneys, spleen, brain or pancreas are detected after inoculation with PMSB. However, there are visible lung metastasis nodules in over 90% hosts of Controls yet no in PMSB groups (Figure 6B). Tumor metastatic dynamics into lung has been collaboratively reversed in ultimate stage by PMSB. Body weight curves of PMSB groups, plotted at three days intervals, keep paralleling closely that of the control group, with no significant differences between them (Figure 6C). All animals have continuously thrived through the experiment, as well as no suppression of innate MSCs/HSCs in bone marrow or indication of autoimmunity observed.

### Effect of NK and T cell depletion on tumor progression

Elimination of NK and CD8^+^ T cells would partly enhance tumor progressive dynamics of PMSB-formulated mice versus mice receiving normal rat IgG as control, whereas the elimination of regulatory CD4CD25^+^ T cells could not enhance tumor growth as compared with control group. The latent progression deterrence by PMSB could be partly terminated by depletion of NK and CD8^+^ T cells but not by the Treg elimination (Supplementary Figure S7A–S7C).

### PMSB impairs tumor cell survival via co-culture

Additional PMSB were incubated with tumor cells at a ratio of 1:120 (spheroids:tumor cells) in DMEM with 10% normal serum for over 4 days of co-culture assay, which shows that survival viability of *in vitro* tumor cells has kept unimpaired in Control group, yet was significantly impaired in CMSB groups, especially in hPSC group (Supplementary Figure S8).

## DISCUSSION

Multiple strategies have been tried to diminish antitumor resistance, but most human cancers remain incurable mainly due to lacking suitable pluripotent therapeutic options to address the constantly evolving CSCs [1–5, 23–26]. Moreover, therapies directed at the amplified pool of terminal cancer cells, but fail to eliminate cancer stem-pool, might induce evolutionary regeneration of tumor cells with more multitherapy-resistant and aggressive heterogeneity via Darwinian survival selection, resulting in tumor relapse and patient ultimate death [7]. Thus, ideal oncotherapy should address both terminal cancer cells and CSCs by slowing down proliferation of differentiated cancer cells, and especially by increasing apoptosis of CSCs [19]. Yet that remains a formidable clinical challenge to date, since CSC-progression is just aimed at evading peripheral immune surveillance via integrative tricks including selective loss of MHC molecules and immunosuppressive microenvironment induction. Current immunotherapies covering anticancer vaccines could stimulate peripheral immune system via MHC-restricted αβT subsets addressing terminal cancer cells, yet unable to address CSC subsets which keep resistant to MHC-restricted anticancer reactivity [27]. Nonetheless, γδT cells could exhibit MHC-unrestricted lytic activity against certain tumor cells, suggesting their potential for tumoricidal therapy. Unfortunately, innate γδT cell repertoire is too limited to eliminate renewable heterogeneous cancer cells.

Depletion of thymus-based central and peripheral immune networks is the crucial mechanism shared by advanced cancer and many other immune-deficiency disorders. Just as endogenous thymus provides superior central-microenvironment for the evolutionary development of renewable T-cell subsets [8], so cancer stem-pool as hotbed for progression, metastasis, multitherapy-resistance or re-evolution of renewable heterogeneous cells [4]. Perhaps, flexible biologics in 3D mode, ameliorated from renewable pluripotent-cells, may provoke central-microenvironment for unique immune renewal aiming at the constantly evolving CSCs [2, 27], maybe leading to complete CSC elimination and cancer cure. However it is hard to trigger thymus from retrogression into integral regeneration via routine procedure, especially for old patients with advanced tumors to extricate from thymic-retrogression background.

In this work, PMSB could drive the withered thymus of senile hosts into evolutionary regeneration and development, with central microenvironment reprogramming endogenous molecule re-expression for γδT-repertoire undergoing rapid renewal of MHC-unrestricted immunocompetence to aim at the constantly evolving CSC subsets. Subsequently, both *in situ* and migrating CSCs were evidently depleted by the PMSB-remodeled γδT-subsets binding and directing into apoptosis. Especially, PMSB-dominated pluripotent tumoricidal-reactivity, just as expected, could lead to ultimate cure of various tumors covering mammary, lung, colon carcinomas and melanoma.

Thus far, there is still no report describing accurate binding and efficient killing of resistant CSCs by pluripotent cells-engineered 3D-biologics. Comprehensive inspections, therefore, are necessary for elucidating anti-CSC microenvironment dynamics in PMSB-dominated tumoricidal-resistance reversal. Cumulative data have revealed that integrative molecule/cell collaboration is involved in immune microenvironment renewal. IFN-γ production, as shown by magnetic bead microarray, is correlated with RANTES/MIG/IP-10 in that depleting these cytokines causes a significant decrease of IFN-γ; where blockade of IFN-γ also elicits an obvious decrease in RANTES/MIG/IP-10. Still, tumor-free induction by PMSB could be mostly terminated by blockade of IP-10, MIG, RANTES and IFN-γ; or could be partly diminished by T/NK-cell depletion. It is well known that macrophage M1 is considered anti-tumor, whereas M2 is pro-tumor and pro-resistance to chemotherapy. FACS has revealed an enhanced level of M1-subset (CD80^+^) versus M2-subset (CD163^+^) in PMSB groups. Particularly, MIG/IP-10 could chemoattract T/NK and M1 by CXCR3 expressed on activated/memory T/NK [33]; whereas RANTES induces γδT migration to tumor sites. Thus, PMSB-dominated tumoricidal-reactivity to refractory CSCs would be substantially enhanced by joint renewal and elaboration among IFN-γ/IP-10/MIG/RANTES molecule and M1/NK/γδT cellular integrative microenvironments.

Since central immune retrogression with peripheral T-subsets depletion is the shared key mechanism among progressive cancer and many immune-deficiencies covering aging procedures and AIDS, our study may illuminate a prospective pathway to resolve current therapeutic dilemma for such disorders via evoking the comprehensive renewal of *in vivo* central-thymus and peripheral immune-microenvironment.

## MATERIALS AND METHODS

### PMSB preparation

Placenta tissues were obtained after informed consent and mechanically minced into approximately 1–2 mm^3^ and digested by collagenase II for 30 min at 37°C. The mononuclear cells were collected and filtered through a 70-mum nylon mesh (BD Biosciences) and separated via percoll density gradient centrifugation to weed out unwanted cells. Then selected cells were suspended in low-glucose DMEM supplemented with 15% FBS, 10U bFGF, 2 m*M* L-glutamine and 0.1 mg Amikacin/ml at an initial seeding density of 5 × 10^5^ cells/ml. Two days later, the nonadhesive cells were removed by washing with serum-free DMEM. Next, media were replaced twice per week for a consecutive 14-day anchorage-screening period in complete isolation media [12, 13, 18]. Flow cytometry and immunofluorescence were used to detect phenotype characteristics of hPSCs for CD29, CD44, CD73, CD90, CD105, CD133, CD166 and CD200. The purified PSC population with positive phenotype was propagated in dynamic suspension with serum-free DMEM/F12/1640-integrated medium until generating more than 125 floating 3D spheroids/ml with active TERT (Supplementary Video S1; Figure 1), these cells were X-ray ameliorated using RS-2000 biological irradiator (www.radsource.com) at 150 Gy so as to keep the cells metabolically alive yet unable to proliferate and renew (Supplementary Figure S6), frozen in one batch and resuscitated as hPSCs-derived PMSB. Via the same procedure, mPSCs-derived PMSB was prepared from term placenta of syngeneic pregnant mice (Supplementary Video S2); bone marrow-derived MSCs prepared by the same formula were used as PMSB counterpart; Common PSCs in adherent culture were X-ray-ameliorated as PMSB controls.

### Inoculation regimen

Research protocol involving animals was reviewed and approved by institute's Animal Care and Use Committee. 4T1 mammary, LL/2 lung, C26 colon tumor and B16 melanoma cell lines were obtained from American Type Culture Collection (ATCC, Rockville, MD) and propagated by *in vitro* passage in DMEM (Gibco BRL, Grand Island, N.Y.) with 10% of FBS (Gibco, Auckland, N.Z.). Syngeneic Balb/c and C57BL/6 senile mice (Supplementary Figure S1) 12 months of age were maintained in air-filtered laminar flow cabinets under aseptic conditions with a 12-h light/dark cycle. Mice were fed with AIN-93M rodent diet and autoclaved reverse-osmosis treated water to ensure proper health and living environments before study initiation, and then each was challenged with 5 × 10^5^ 4T1, LL/2, C26 or B16 tumor cells subcutaneously into right flank. Seven days later, the tumor challenged-hosts were inoculated subcutaneously with 2 × 10^5^ PMSB cells (m−/h− PSCs-derived or bMSCs-derived PMSB) into left flank. Primary inoculation was followed by prime-boost (2 weeks apart) protocol of week 0–2. These hosts were kept under careful observation for any experiment-associated adverse effects and tumor formation/retrogression. Tumor volume (mm^3^) was determined by the formula: 0.52 × length (mm) × width^2^ (mm)^2^ and plotted at three-day intervals.

### Dynamics assay for CSCs-floating 3D spheroids

The floating-spheroid number reflects the quantity of stem cells capable of self-renewal [7, 30]. Cells isolated from *in vivo* tumor tissues of corresponding groups were propagated via dynamic suspension in serum-free DMEM/F12/1640-integrated medium to generate renewable 3D-spheroids. Subset progressive dynamics, covering renewable/evolutionary diversity and tumorigenesis property, was analyzed according to spheroids regenerating various evolutionary modes under the microenvironment pressure from 1640/F12/2%-FBS selective medium. Immunofluorescence and western blotting were used to detect the expression changes of TERT/Wnt/β-catenin pathway.

### Magnetic bead microarray and western blotting

Non-necrotic fresh tumors or tumor-free local inoculum and thymus were cut into small pieces, homogenized in liquid nitrogen, emulsified by ultrasonication, extracted with ice-cold RIPA lysis buffer at a ratio of 100 mg:1 ml, and then passed through a fine mesh sieve (Bellco Glass). The extracted total protein was concentrated to 12 mg/ml and then detected for peripheral molecules covering IP-10, MIG, RANTES, IFN-γ, IL-4 and VEGF expression using Milliplex Magnetic Bead microarray (Millipore, MA, US) with Luminex 200 analysis system (Luminex Corp., Austin, TX). Thymosin and β-catenin levels were determined by western blotting using 50 μg extracted proteins from each sample.

### *In vivo* blockade of IFN-γ/RANTES loop

Additional tumor-challenged/PMSB-inoculated hosts were i.p. injected with neutralizing antibodies specific for IFN-γ (clone XMG1.2, Bioexpress), IP-10/CXCL10, MIG/CXCL9 and CCL5/RANTES (clone 33036.211, clone 49106.11 and clone 53405.111, R&D Systems, Minneapolis, MN) on day −8, −4, −1, 0, 1, 4, 8 as previously reported [31–35], with normal rat IgG (Sigma Chemical Co) injected according to the same protocol (100 μg/100 μl of PBS) as control. The tissue level blockades were confirmed by Luminex 200 analysis system.

### Protective responses assay of posterior thymopoiesis versus CSC-subsets

Dual-Color IFN-γ/IL-4 ELISpot Kit (R&D Systems# ELD5217) was adopted for monitoring cross-protective responses of PMSB-inoculated hosts to CSC subsets. Briefly, thymocytes were harvested from host thymus 2 weeks after the boost. 5 × 10^4^ recipient thymocytes as responder cells and 3 × 10^3^ PMSB cells, or tumor spheroid cells as stimulators were respectively performed according to the manufacturer's protocol with common PSCs as stimulator control. Spots were automatically scanned and enumerated using ELISpot plate reader (Cellular Technology Ltd., Cleveland, OH).

### Histomorphometric and FACS assay

Tumor specimens with adjacent tissues, relevant internal organs and inguinal sentinel lymph nodes (SLN) were harvested and fixed in 4% neutral buffered formalin solution for paraffin sectioning and staining. Immunohistochemical test for *in situ* detection were performed with anti-MUC-1 (BD), CD49b/NK1.1 (Biolegend), anti-CD4, CD8, CD25, Foxp3, β-catenin, TERT (all abcam), and anti-CD44, CD133 (all Chemicon) monoclonal antibody severally. Imaging of thymic lobules for renewal hotspots were captured using Leica scanning confocal microscope. Thymic and tumor tissue/cells were stained with thymosin-α1(abcam)/-β4(Millipore) for immunofluorescence or with TCRαβ, TCRγδ, CD3, CD38, CD80, CD163, CD45RA (all BD) for positive subsets via FACS-Aria III cell sorting system. Successive subsets were incubated with 3D-CSC-subsets from *in vivo* tumor at a ratio of 120:1 (thymocyte: spheroid) in DMEM/F12 with 2% normal serum for over 72 hours of reactivity between renovated T-subsets and CSC pool.

### Statistical analysis

Experimental data were subjected to one-way ANOVA plus Tukey post-hoc test. The tumor-free survival was analyzed using log rank test and Kaplan-Meier method. Tumor cell for TERT/MUC-1/CD44/CD133 expression index and lymphocyte for NK-1, TCRαβ, TCRγδ, CD3, CD38 or CD45RA were respectively calculated as a ratio of the positive cell number to the total tumor cell/lymphocytes number based on the mean value from ten high-power fields via computer-assisted assay. *P* values < 0.05 were considered significant.

Supplementary Material: Supplementary Figures (S1–S8) are available in the online version of the paper.

## SUPPLEMENTARY FIGURES AND VIDEOS



## Abbreviations

MHC: major histocompatability complex
CSCs: cancer-sustaining stem cell subset
PSCs: placenta-based somatic stem cells
PMSB: PSC-in-3D multipotent spheroid-ameliorated biologics
CB: common non-3D biologics
TERT: telomerase reverse transcriptase
FITC: fluorescein isothiocyanate
FACS: fluorescence-activated cell sorting
ELISpot: enzyme-linked immunospot assay
