# Supplementary material for: A biotherapy based on PSCs-in-3D spheroid-ameliorated biologics depletes in vivo cancer-sustaining stem cells
Source: Oncotarget. 2015 Oct 19;6(38):40762–74. doi: 10.18632/oncotarget.5691 (PMC4747367; doi:10.18632/oncotarget.5691)
Supplement: Supplementary file 1 [file oncotarget-06-40762-s001.pdf]

SUPPLEMENTARY FIGURES AND VIDEOS

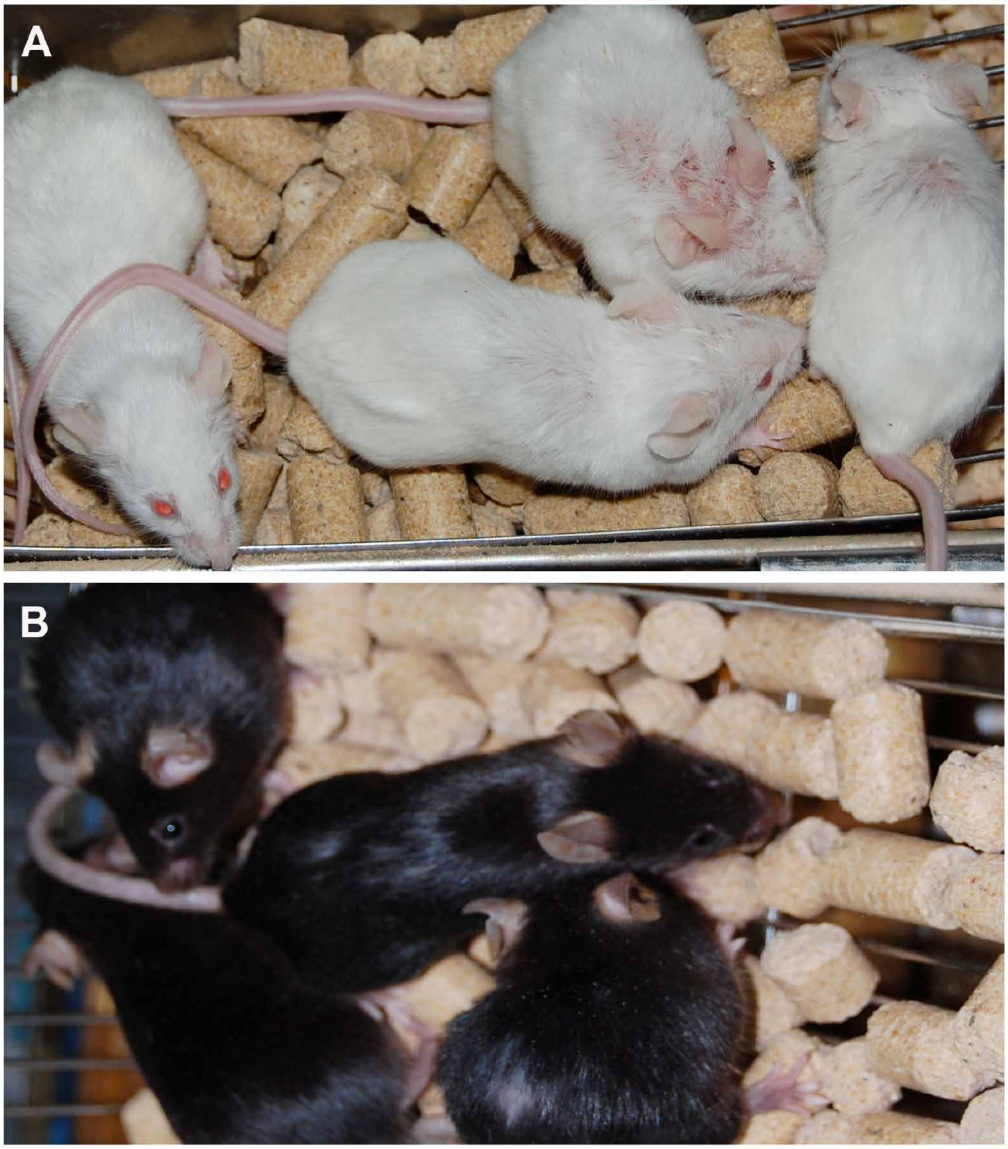

Supplementary Figure S1: Experiment animal preparation. Research protocol involving animals was reviewed and approved by institute's Animal Care and Use Committee. Syngeneic Balb/c **A.** and C57BL/6 **B.** mice 12 months of age (with senile thymus source) were used as tumor-challenged hosts in this study. Mice were fed with AIN-93M rodent diet and autoclaved reverse-osmosis treated water to ensure proper health and living environments before study initiation.

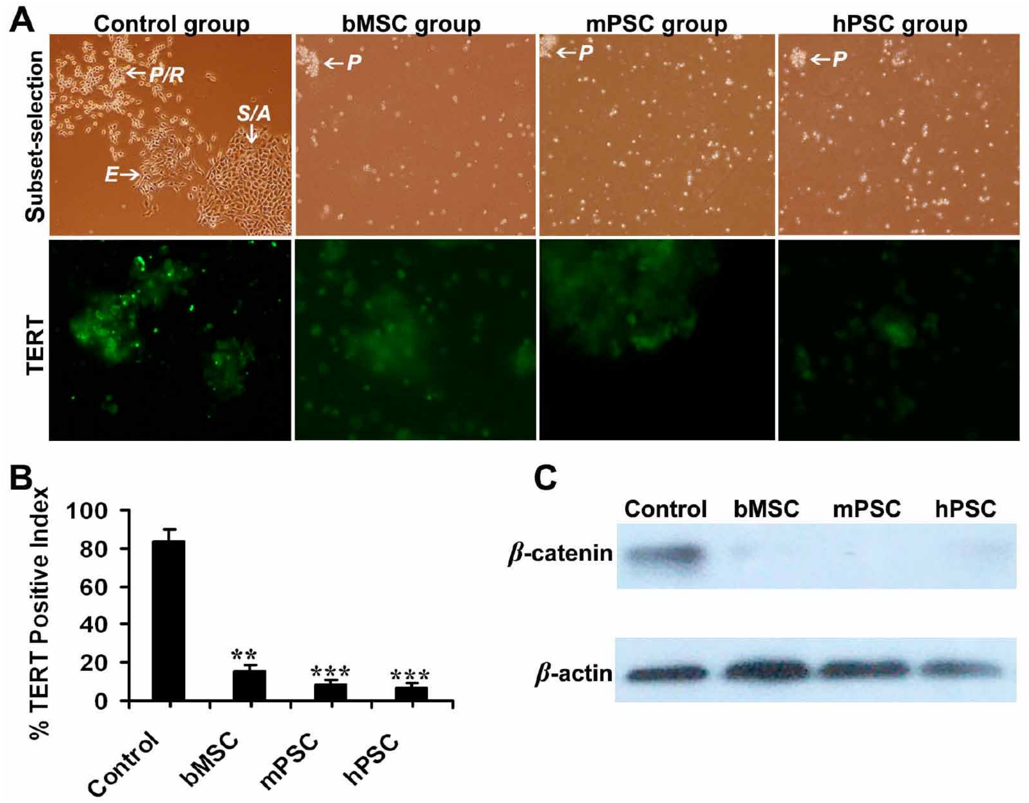

Supplementary Figure S2: Blockade of TERT/Wnt/β-catenin loop of CSC subsets. A. Panel 1: CSC subsets from Control group readily re-generate hierarchical lineages including primary/renewing (*P/R*), evolving (*E*) and selected/amplifying (*S/A*) clones (subset progression/evolution). However, the subset-evolution properties have inevitably lost in PMSB groups. Immunofluorescence (Panel 2) manifested that activatory TERT, just as shown in control group, has declined evidently or lost in three PMSB groups. Latent renewal, self-selection and heterogeneous transitions of CSC subsets have been synchronously deterred by PMSB. **B.** TERT positive index of CSCs was calculated as a ratio of positive cell number to total cells, which detected numerous TERT positive cells in control group, yet only a very few such positive tumor cells in PMSB groups with about 1/12 positive index of control group (*$P < 0.05$; **$P < 0.01$; ***$P < 0.005$ versus control groups). **C.** Levels of total β-catenin were determined by western blotting using the extracted proteins from each sample. Active β-catenin has collectively lost due to self-renewal loss of CSCs under PMSB-triggered immune microenvironment-renewal.

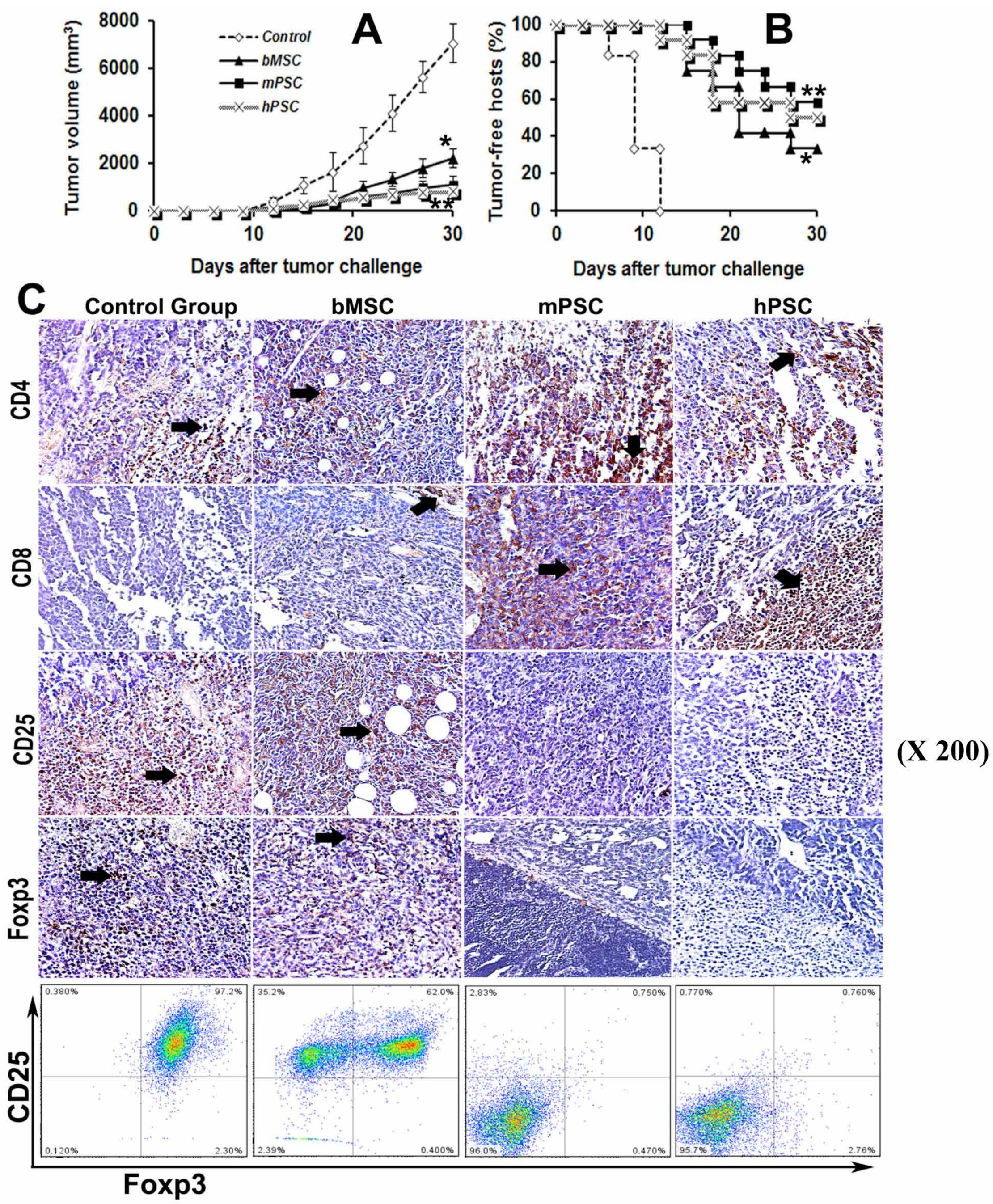

Supplementary Figure S3: Integrative resuscitation of peripheral immune microenvironment with tumor retrogression under PMSB-triggered quick microenvironment-renewal. A. Tumor growth suppression rate in three PMSB-inoculated groups remains over 71% and 68% respectively at 3 weeks and one month after tumor challenges. The tumor progression was evidently deterred in PMSB groups, especially in PSCs groups. **B.** PMSB groups keep about 58.3% ~ 50% of tumor-free survival after study termination. In contrast, all hosts in Control group exhibited tumor genesis prior to day 12 post-tumor challenges. The above data are cumulative results of four separate experiments and expressed as percentage of tumor-free mice at the indicated time points. **C.** Peripheral immune microenvironment assay shows that there are plentiful $CD8^+$ T and $CD4^+$ T cells ($CD25^-Foxp3^-$) scattering in remnant tumor nests and stroma in PMSB groups, especially in PSC groups. Whereas numerous $CD25^+Foxp3^+$ T cells were congregated in tumor stroma of Control group, yet almost no such T suppressor cells in PSC groups. FACS assay manifested T cells recruited to tumors of control group bearing an elevated expression index for both CD25 and Foxp3 ($CD25^+Foxp3^+$ $T > 97\%$) versus PMSB groups ($CD25^+Foxp3^+$ $T < 1\%$ in PSC groups). It just shows integrative resuscitation of peripheral immune microenvironment following central immune renovation by PMSB with sequent tumor retrogression (*$P < 0.05$; **$P < 0.01$ versus control groups).

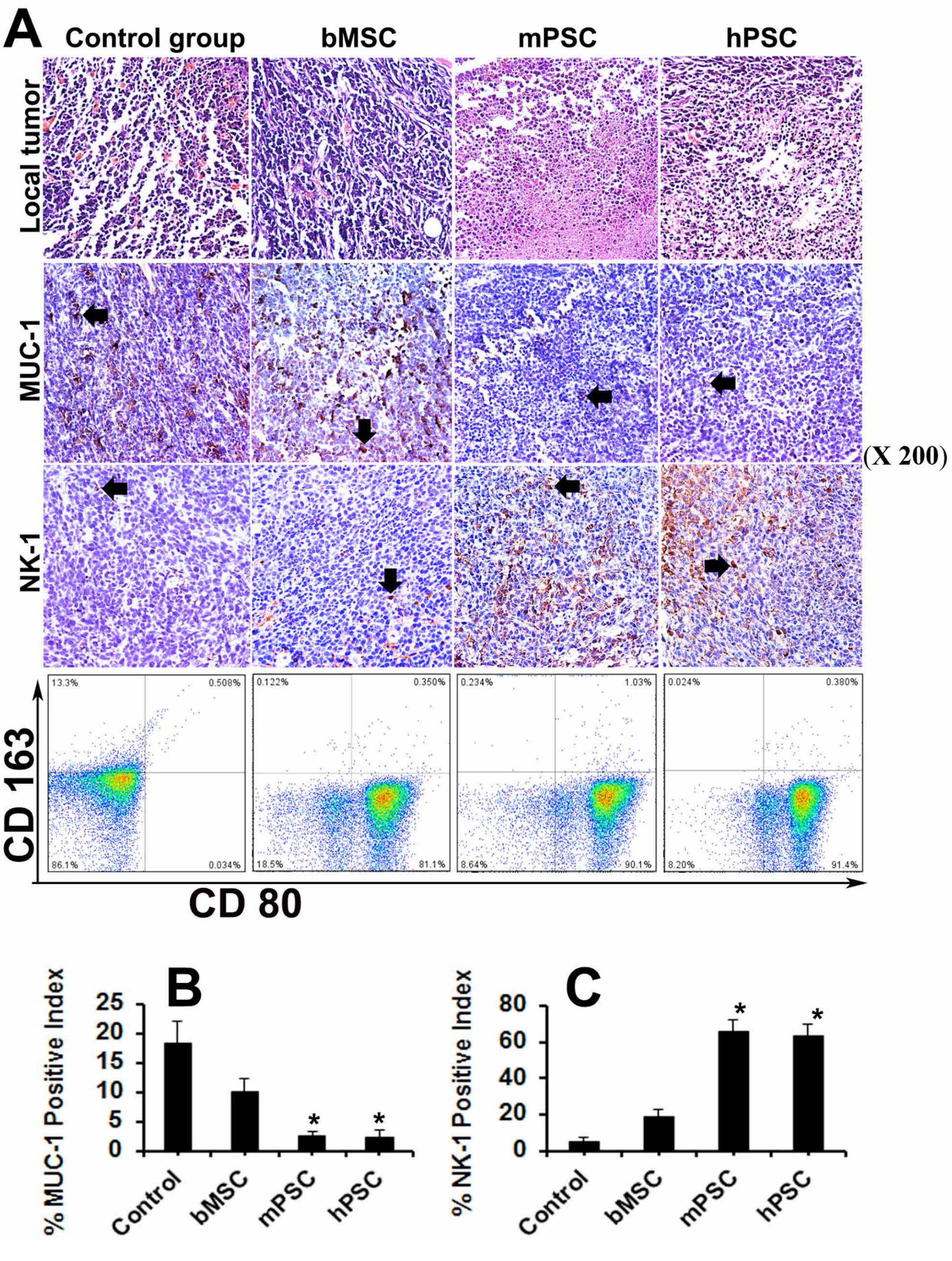

Supplementary Figure S4: *In situ* CSC subsets were depleted by PMSB. **A.** *Panel 1*: The tumor cells in control group have kept active proliferation around newborn blood vessels. In contrast, remnant tumor cells in three PMSB groups were detected undergoing retrogression, cytolysis and pyknosis, with extensive macrophage infiltration and lower angiogenic progression. *Panel 2*: plentiful MUC-1$^+$ *in situ* CSCs (arrow) were found to scatter throughout the tumor nests in control group, yet only several such cells were scattered in PMSB groups, especially, almost no such cells surviving in PSCs groups. *Panel 3*: there were very few CD49b/NK-1$^+$ lymphocytes (activated NK) infiltrating the tumor tissue of control group; whereas numerous CD49b/NK-1$^+$ cells (arrow) were detected to congregate around or infiltrate into the remnant tumor cells in PMSB groups. *Panel 4*: FACS just revealed an enhanced level of CD80 (M1 subset, >81%) versus CD163 (M2 subset, <1%) for the macrophages recruited to tumors in PMSB groups, yet the M1/M2 ratio has fallen to around zero in control group. **B.** Tumor cells MUC-1 positive index was calculated as a ratio of positive cell number to total tumor cells via histomorphometric assay. As shown in the graph, the assay verified that there were numerous MUC-1 positive tumor cells in control group, yet only few such positive tumor cells in PMSB groups, especially in PSCs groups, with about 1/9 ~ 1/10 positive index of control group. **C.** NK-1 positive index in PMSB groups has increased about fivefold over Controls. Values are expressed as average positive index ± SD (*P < 0.01).

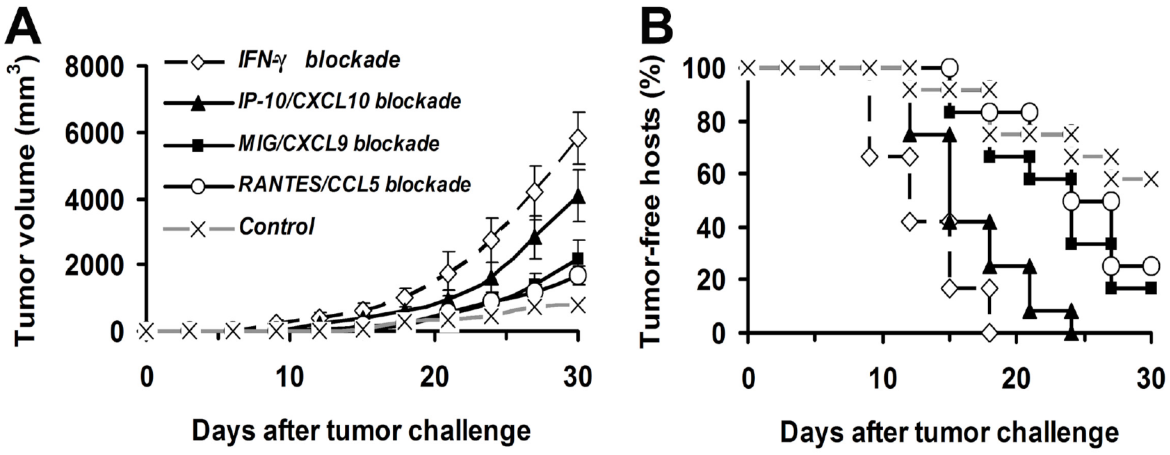

Supplementary Figure S5: IFN-RANTES axis blockade enhances tumor progressive dynamics in PMSB-inoculated hosts. Additional tumor-challenged/PMSB-inoculated hosts were established and injected with neutralizing antibodies specific for IFN-γ, IP-10/CXCL10, MIG/CXCL9 and RANTES/CCL5 to blockade IFN-γ/IP-10/RANTES axis, with normal rat IgG injected according to the same protocol as control. **A.** Study shows that the blockade of key factors in IFN-γ/RANTES defense loop covering IFN-γ, IP-10/CXCL10, MIG/CXCL9 and RANTES/CCL5 could boost tumor progressive dynamics of PMSB-inoculated hosts ($P < 0.05$ versus controls). **B.** Hosts subjected to IFN-γ blockade exhibited tumor establishment between day 9 and 18 post-tumor challenge. Hosts subjected to IP-10/CXCL10 blockade exhibited tumor establishment between day 12 and 24 post-tumor challenge; however, 16.67% and 25% hosts subjected to MIG/CXCL9 and RANTES/CCL5 blockades kept tumor-free versus 58.3% hosts in controls keeping tumor-free. Tumor-free induction by PMSB were mostly diminished by IFN-γ or IP-10/CXCL10 blockade and evidently deterred by MIG/CXCL9 or RANTES/CCL5 blockade ($P < 0.05$ versus controls). Cumulative results of four separate experiments are expressed as percentage of tumor-free hosts at the indicated time points.

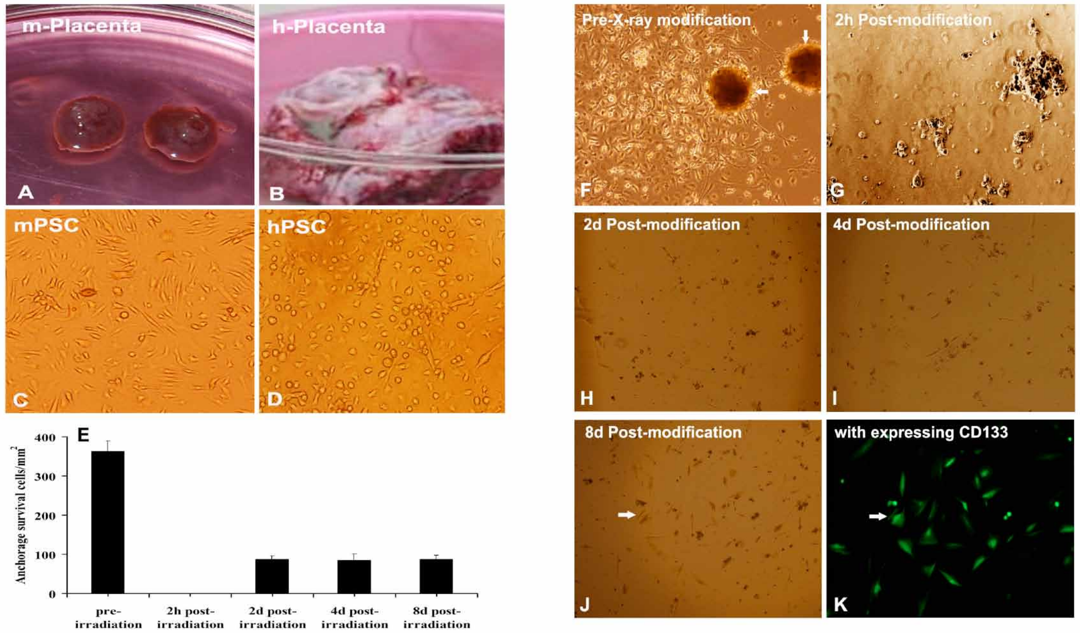

Supplementary Figure S6: Fresh term placenta and their offspring PSCs with dynamic feature after X-ray Modification. A. Fresh placenta of mice appears to be in a pocket mooncake-shaped pattern. **B.** A block of the human fresh placenta. **C.** Phase contrast imaging of mPSCs (from murine placenta) at population doubling 2 exhibits a moderate proliferation of short spindle-shaped cells arranged in a loose basket-weave pattern. **D.** Phase contrast imaging of population doubling 2 hPSCs (from human placenta) exhibits a mild proliferation of short spindle-shaped cells loosely arranged in a basket-weave pattern (before serum-free suspension culture). **E.** After X-ray modification, 3D hPSCs have fully lost self-replication/renewal potential and could be used as safe PMSB biologics. Before X-ray modification the purified hPSCs were expanded and engaged in 3D multicellular floating-spheroid transition (**F.** arrows) with self-replication/renewal potential in serum-free suspension condition. The PSC populations were X-ray modified with 150 Gy so as to keep the cells metabolically alive yet unable to proliferate for safe PMSB preparation. 2 hours after X-ray modification the PSCs revealed an acute irradiative shock by merging themselves into each other **G.** 2 days later **H.** some PSCs gradually unmerged and embarked on a revivification yet non-proliferation. 4 and 8 days later **I–J.** some PSCs remained survival yet without growth, and meanwhile expressing CD133 (**J–K.** arrows indicate the same field).

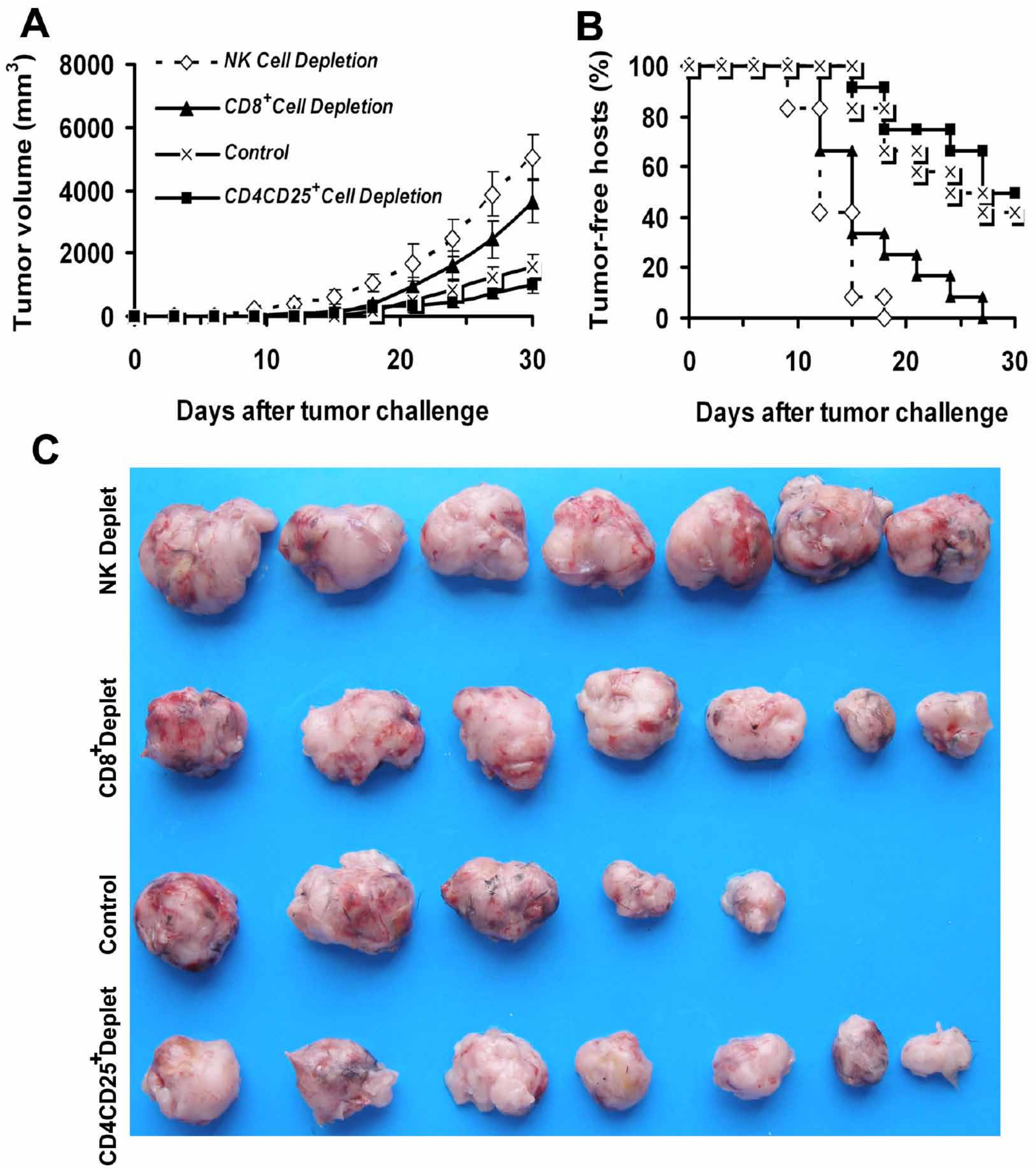

Supplementary Figure S7: NK/T depletion affects tumor progressive dynamics of PMSB-inoculated hosts. Additional tumor-challenged/PMSB-inoculated hosts were established and injected with anti-asialo GM1 antiserum, or rat mAbs of anti-CD8, anti-CD4/-CD25, with normal rat IgG as control. **A.** Graph shows that both NK and CD8 T cells depletions could enhance tumor development ($P < 0.05$ versus controls), yet the elimination of regulatory CD4CD25$^+$ T cells could not ($P > 0.05$). **B.** All hosts in NK and CD8 depletion groups exhibited tumor establishment prior to day 18 and 27 post-tumor challenges. In contrast, over 40% hosts in CD4CD25$^+$ cell depletion kept tumor-free post-tumor challenge. Tumor-free induction by PMSB was partly terminated by NK or CD8 elimination. Cumulative results of four separate experiments are expressed as percentage of tumor-free hosts at the indicated time points. **C.** As the photograph shows representative specimens from NK/T depletion and Control groups, NK-depletion group (top panel) exhibited tumor nodules faster-growing than other groups. CD8-depletion group exhibited similar tumor growth like NK-depletion group (middle-upper panel); whereas tumor establishment and growth continued to remain delayed or deterred in Control (middle-lower panel). Unlike NK or CD8 depletion, the CD4CD25$^+$ T cell elimination could not boost tumor growth (bottom panel, $P > 0.05$ versus Control).

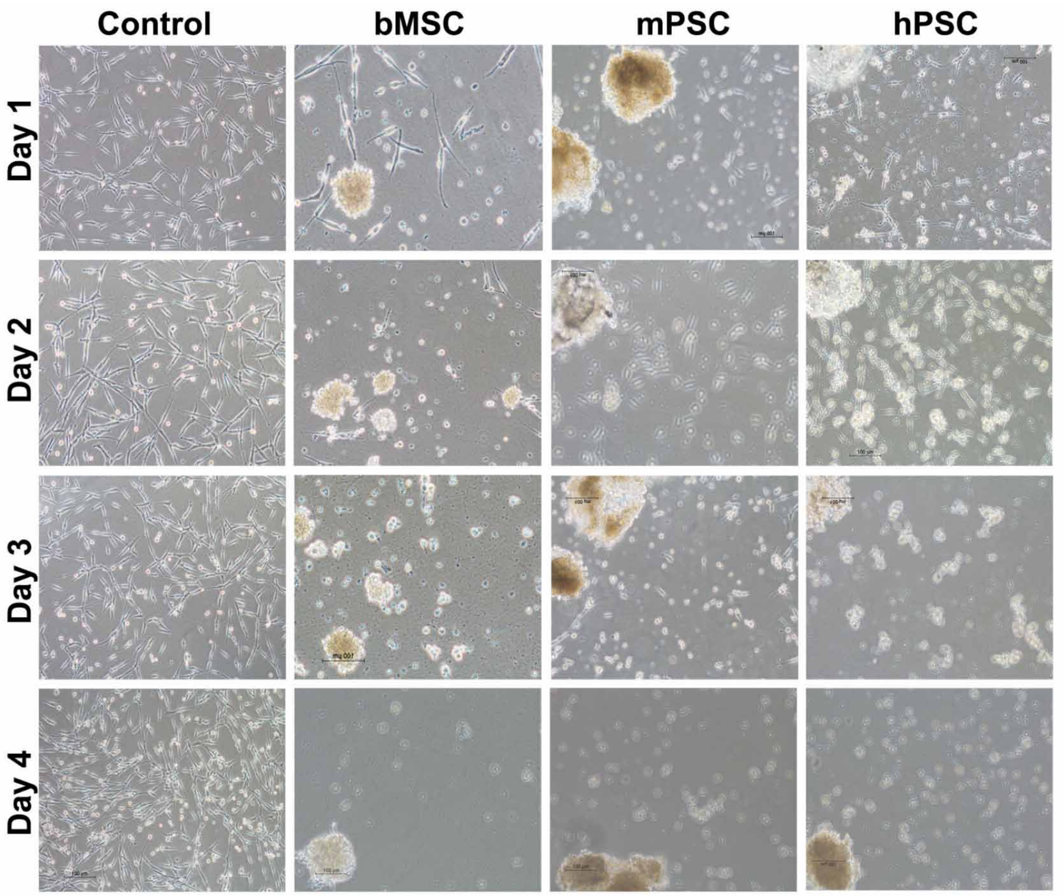

Supplementary Figure S8: Effects of PMSB/tumor cells co-culture on *in vitro* tumor cell survival viability. Additional PMSB were incubated with tumor cells at a ratio of 1:120 (spheroids:tumor cells) in DMEM with 10% normal serum for over 4 days of *in vitro* co-culture assays, which shows that survival viability of *in vitro* tumor cells has kept unimpaired in Control group, yet was significantly impaired in CMSB groups, especially in hPSC group.

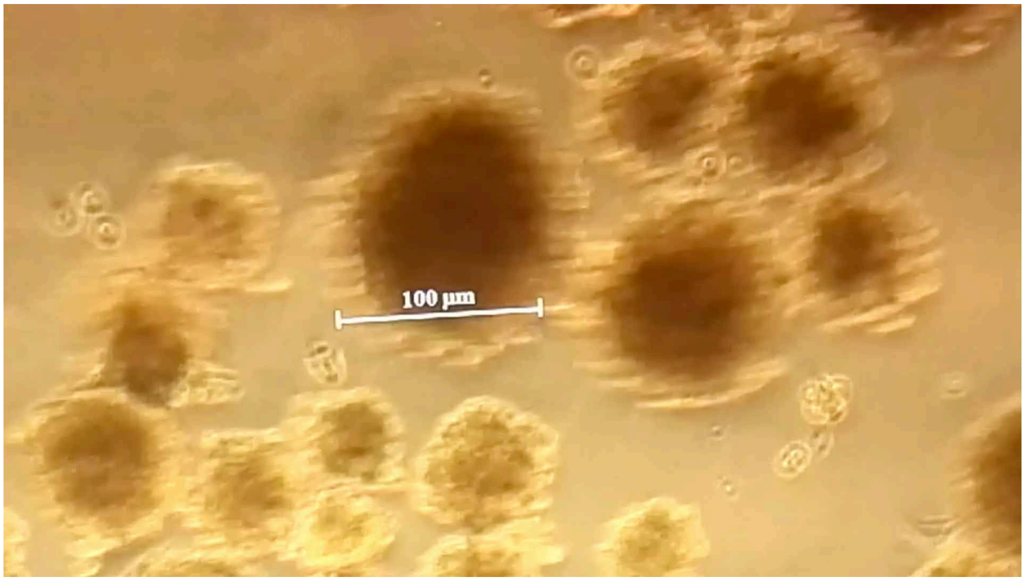

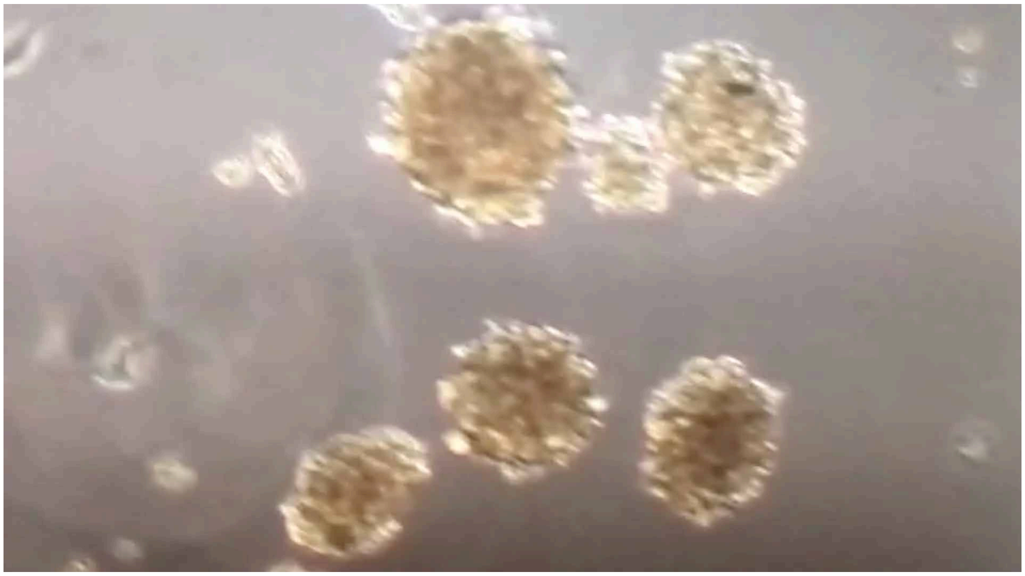

Supplementary Video S1–S2: Since the purified h-/m-PSCs possess sufficient anchorage viability and even keep plate-adhering under the serum-free culture condition, the cells have to be propagated and unattached by dynamic shaking suspension model to regenerate floating-3D renewable spheroids, where each spheroid contains more than 220 stem cells with each dimension about 100-120µm for synchronous multiepitope-integration. More than 125 3D-floating spheroids could be enriched per ml for about 20 days of dynamic shaking suspension propagation. Routine cultures could not generate 3D-spheroids.
